# Comparative Assessment of Nitrogen Concentration Effect on Microalgal Growth and Biochemical Characteristics of Two *Chlorella* Strains Cultivated in Digestate

**DOI:** 10.3390/md20070415

**Published:** 2022-06-25

**Authors:** Savvas Giannis Mastropetros, Eleni Koutra, Mohammed Amouri, Majda Aziza, Sameh Samir Ali, Michael Kornaros

**Affiliations:** 1 Laboratory of Biochemical Engineering & Environmental Technology (LBEET), Department of Chemical Engineering, University of Patras, 26504 Patras, Greece; savvasgiannismas@gmail.com (S.G.M.); ekoutra@chemeng.upatras.gr (E.K.); 2 Centre de Développement des Energies Renouvelables (CDER), BP. 62, Route de l’Observatoire, Algiers 16340, Algeria; amourimed21@yahoo.fr (M.A.); majdaaminaaziza@gmail.com (M.A.); 3 School of the Environment and Safety Engineering, Biofuels Institute, Jiangsu University, Xuefu Road 301, Zhenjiang 212013, China; 1000004956@ujs.edu.cn; 4 Botany Department, Faculty of Science, Tanta University, Tanta 31527, Egypt

**Keywords:** digestate, *Chlorella vulgaris*, local isolate, ammonia stripping, bioremediation, biomass composition, pigments, fatty acid profile

## Abstract

Microalgae have been recently recognized as a promising alternative for the effective treatment of anaerobic digestion effluents. However, to date, a widely applied microalgae-based process is still absent, due to several constraints mainly attributed to high ammonia concentrations and turbidity, both hindering microalgal growth. Within this scope, the purpose of the present study was to investigate the performance of two *Chlorella* strains, SAG 211-11b and a local Algerian isolate, under different nitrogen levels, upon ammonia stripping. The experiments were performed on cylindrical photobioreactors under controlled pH (7.8 ± 0.2) and temperature (25 ± 2 °C). Cultures were monitored for biomass production and substrate consumption. After sampling at the beginning of the stationary phase of growth (12th day) and after the maturation of the cells (24th day), an analysis of the produced biomass was conducted, in terms of its biochemical components. The local isolate grew better than *C. vulgaris* 211-11b, resulting in 1.43 mg L^−1^ biomass compared to 1.02 mg L^−1^ under 25 mg NH_4_-N L^−1^, while organic carbon and nutrient consumption varied between the two strains and different conditions. Concerning biomass quality, a high initial NH_4_-N concentration led to high protein content, while low nitrogen levels favored fatty acid (FA) accumulation, though the production of pigments was inhibited. In particular, the protein content of the final biomass was determined close to 45% of the dry weight in all experimental scenarios with adequate nitrogen, while proteins decreased, and the fatty acids approached 20% in the case of the local isolate grown on the substrate with the lowest initial ammonium nitrogen (25 mg NH_4_-N L^−1^). The novelty of the present work lies in the comparison of a microalga with industrial applications against a local isolate of the same species, which may prove to be even more robust and profitable.

## 1. Introduction

Currently, the application of anaerobic digestion for wastewater treatment and biogas production is expanding. According to the Global Bioenergy Statistics 2020 of the World Bioenergy Association, 59.3 billion m^3^ of biogas was produced globally in 2018, whilst it is predicted that biogas needs will reach 29.5 GW in 2022 [1]. The excess biogas production through anaerobic digestion results in a dramatic increase in effluents, called digestates. This type of by-product is enriched with remarkable levels of nitrogen, phosphorous and organic substances depending on the composition of the substrates used [2]. After an analysis of 213 different liquid digestates, an average ammonium nitrogen concentration was determined close to 2 g L^−1^, forming a C:N ratio of around 6.5, while phosphorus oxide levels were 0.39% *w*/*w*, based on digestate fresh weight [3]. Although digestates can be used as efficient fertilizers and soil conditioners, they can be harmful to natural ecosystems due to their high ammonia and organic content, resulting in eutrophication phenomena in aquatic ecosystems [4,5]. Moreover, anaerobic effluents may contain critical amounts of sulfides and arsenic, up to 1.9 and 0.07 mg L^−1^, respectively, with potentially toxic effects on flora and fauna [6]. Therefore, the effective management of anaerobic digestion effluents prior to disposal represents a critical challenge [7]. However, physicochemical treatment for reducing digestates’ pollutants seems to be significantly costly and unsustainable. In contrast, biological processes are under investigation in order to provide an effective and environmentally friendly solution to digestate management [8].

Within this scope, microalgal cultures are considered as a promising alternative to common digestate treatment methods, as they effectively consume inorganic and organic components provided by effluents [9]. In addition, the harvested biomass usually contains high amounts of lipids, polysaccharides, proteins and other secondary metabolites, including pigments, vitamins and phenolic compounds, that are characterized by high antioxidant activity [10,11]. Microalgae-based polyunsaturated fatty acids (PUFAs) include eicosapentanoic acid (EPA), docosahexaenoic acid (DHA) and other nutritionally important omega-3s, while the neutral triacyl-glycerides (TAGs) are more suitable for transesterification and biodiesel production [12]. Furthermore, carbohydrates found either in intracellular plastids as energy storage materials or in the microalgal cell wall could be exploited for bioethanol production [13]. Finally, microalgal proteins are characterized by high nutritional value, due to the high variety of included amino acids [14]. Despite the remarkable microalgal performance in digestates, there are still important obstacles that hinder process scale-up, including high operating costs, inefficient harvesting and the sensitivity of many species to culture conditions [15]. Interestingly, both total nitrogen and phosphorus can be totally consumed, but the Chemical Oxygen Demand (COD) reduction ranges from 70 to 90%, as previously reviewed [16], showing that not all the organic load is biodegradable.

The use of low-cost substrates, such as digestates, coupled to microalgae biorefinery could enhance the profitability of microalgal cultivation [17]. However, high nitrogen levels remain a challenge for effective microalgal growth. In general, ammonia dissolved in the growth medium is more harmful for microalgae cells than ammonium cations [18]. Moreover, studies argue that toxicity tolerance levels vary from species to species [19,20]. Indicatively, the growth of *Chlorella* sp. in a food waste anaerobic effluent, which contained 3.8 g NH_4_-N L^−1^, was severely inhibited as a function of the illuminance intensity and the turbidity of the digestate provided, due to the cells’ requirements for light [21]. Furthermore, the productivity of an outdoor microalgae consortium was reduced by 21%, when the ammonium concentration of a piggery-originated digestate was doubled from 0.8 to 1.6 g L^−1^. Thus, CO_2_ supplementation was necessary to alleviate this effect [22].

Therefore, several pretreatment methods are usually applied prior to digestate use, in order to reduce effluent toxicity and promote microalgal growth. Struvite precipitation is also a common wastewater treatment method to mainly recover soluble phosphorus, after magnesium addition. Although up to 90% phosphorus recovery can be observed through this process, the removal of the potentially harmful ammonium nitrogen cannot exceed 40% [3]. Targeting more satisfactory nitrogen recovery, exceeding 90%, membranes can be used for the micro-, ultra- and nano-filtration of kitchen and food waste digestate [23]. However, despite the great recovery efficiency, membranes’ operational costs should be reduced [3,23]. Moreover, a variety of adsorbents have been studied regarding their ability to remove ammonium from digestates. Zeolite, which is the most common, has a capacity of 19 g NH_4_^+^ kg^−1^, but it is not characterized by selectivity and pores are often blocked by ions, such as Mg^2+^ and Ca^2+^ [24]. Interestingly, ammonia stripping has been successfully applied to piggery wastewater, agricultural effluents and landfill leachate [25,26,27]. However, regarding digestates, though it is possible to remove up to 90% ammonia [28], it is necessary to estimate all the factors that affect its optimization. Ammonia stripping is a physicochemical procedure that allows the conversion of ammonium cations to ammonia gas dissolved in water, which can be subsequently removed from the liquid phase through aeration. Since the ammonia–ammonium equilibrium depends directly on the temperature and pH of the medium [29], an increase in both pH and temperature is highly recommended in order to achieve a satisfactory ammonia removal rate. Continuous ammonia stripping experiments on digestate have previously shown that an air to liquid ratio of 1850 and pH of 10.5 induced 92.8% ammonium removal. In contrast, a temperature increase from 30 °C to 70 °C did not notably affect the process [28]. Lastly, ammonia recovery is performed by a strong acid trap, which prevents ammonia from being released into the atmosphere. Instead, ammonium salts can be formed that can be used as soil conditioners and fertilizers [30].

In recent years, considerable research interest has also been attracted in the isolation and identification of native species of microalgae and cyanobacteria. Local isolates could be found in different habitats, in salt water, freshwater and several types of wastewaters. In addition to microscopy methods, molecular markers are also used for the identification procedure (e.g., 18S rDNA and ITS genes) [31]. As the strains found in nature are likely to exhibit rare characteristics due to long-term adaptation to harsh conditions, more studies are needed for their performance on tested substrates. Local isolates may outperform their conventional competitors in bioproducts’ recovery applications such as the preparation of high-protein food supplements and biodiesel production from fatty acids [31,32].

Within this scope, the purpose of the present study was to evaluate the performance of two strains of the industrial microalgal genus *Chlorella,* on a digestate derived from the anaerobic digestion of a mixture of expired food products (meat, bread, fruit and vegetables) and hydrolysate of used disposable nappies. Such substrates are challenging due to their high ammonium nitrogen concentration, which can inhibit photosynthesis [33]. A comparison between *C. vulgaris* (SAG 211-11b) and an Algerian isolate was made regarding cultivation under different initial concentrations of ammonium nitrogen, applied after ammonia stripping. The comparison of already exploited microalgae with new strains, which were locally isolated, is an important contribution in the international literature, as it leads to the expansion of the current knowledge. Furthermore, digestate remediation and the potential use of the produced biomass were evaluated, in order to determine the most effective method for the concomitant digestate treatment and valuable biomass production.

## 2. Results and Discussion

### 2.1. Nutrients and COD Consumption

Both *Chlorella* strains, 211-11b and the local isolate, were cultivated in digestate with defined nutrient concentrations after ammonia stripping, as described in Table 1. The applied stripping process affected the digestate characteristics, causing slight alterations in COD and total phosphorous (TP) content, especially in the case of 25 mg L^−1^ of initial NH_4_-N concentration, as it required the longest period in the stripping column (3 days). However, the consumption of nutrients during the microalgal growth was of greater interest. Firstly, the initial COD values of cultures decreased the longer the ammonia stripping process lasted. The COD removal did not significantly depend on the cultivated strain, but on the amount of NH_4_-N initially provided in the culture. The COD removal was notably lower for the cultures with the limiting amount of NH_4_-N and did not exceed 60%. *C. vulgaris*, when tested on piggery wastewater, decreased COD levels by 99%, corresponding to 480 mg L^−1^ removal [34], while in the present study, a COD reduction of approximately 3 g L^−1^ was observed. Concerning NH_4_-N, the greatest nitrogen removal was observed in the experiments with the minimum initial nitrogen concentration and no statistically important difference between the two strains was detected. The nitrogen-sufficient *C. vulgaris* 211-11b cultures showed around 100 mg NH_4_-N L^−1^ removal, in contrast to the local isolate cultures that exhibited even 193 mg L^−1^ reduction. Comparable results (up to 240 mg L^−1^) were reported in *C. vulgaris* cultures with paper-derived digestate [35]. Moreover, cultivation of *C. vulgaris* in diluted digestate of piggery waste and corn caused the removal of around 100 mg NH_4_-N L^−1^ [36]. Nitrogen became the limiting nutrient for the growth of both strains, only at an initial concentration of 25 mg L^−1^. In contrast, almost complete phosphorous removal was observed in all tested cultures, in accordance with previous studies regarding pulp and paper digestate with TP of 8 mg L^−1^ [35], while *C. vulgaris* can demonstrate phosphate removal of up to 30 mg L^−1^ [36]. In the present study, at a nitrogen surplus, 500 mg NH_4_-N L^−1^, microalgal growth was probably restricted either by the limiting light penetration or the complete consumption of the biodegradable organic matter.

### 2.2. Microalgal Growth under Different Initial Concentrations of Ammonium Nitrogen

**Figure 1:**
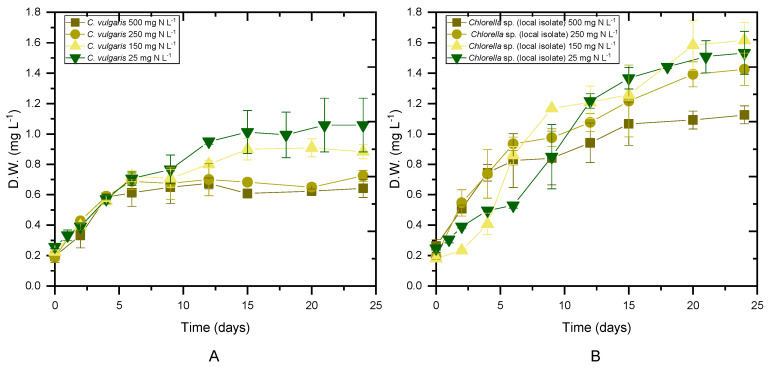
Biomass growth as a function of time for different initial concentrations of NH_4_-N. ((**A**) Biomass of *C. vulgaris* 211-11b, (**B**) biomass of local isolate *Chlorella* sp.).

Both *Chlorella* strains were cultivated in digestate that had previously been stripped of NH_4_-N to evaluate the impact of initial ammonium content on microalgae performance. For each experimental scenario, biomass production, productivity and the maximum specific growth rate (μ_max_) were determined, as shown in Table 2. The average values of the biomass concentration during the stationary phase (Figure 1) of the cultures are presented as the maximum biomass achieved. Biomass production seemed to depend on both the used strain and the qualitative characteristics of the substrate (*p* < 0.05). Examining the cases per strain, the local isolate showed a higher concentration of biomass compared to the *C. vulgaris* 211-11b in untreated digestate, while no statistical difference was found between the two strains in cultures with 25 mg NH_4_-N L^−1^. The highest biomass accumulation was observed in cases of initial 25 mg NH_4_-N L^−^^1^. In addition, the local isolate was characterized by higher productivity in both untreated digestate and in nitrogen-limiting substrates. Unfortunately, the behavior of the results for the intermediate concentrations of NH_4_-N was not clear after the statistical analysis. More repetitions of the same experiment would make it easier to draw conclusions. Both strains yielded close to 0.1 g L^−1^ day^−1^ under all tested conditions (Table 2). With a decreasing initial NH_4_-N concentration, a decrease in the maximum specific growth rate was observed, resulting in μ_max_ values of 0.15 and 0.13 d^−1^ for *C. vulgaris* 211-11b and the local isolate, respectively, under 25 mg NH_4_-N L^−1^. When nitrogen was not the limiting growth factor, the maximum specific growth rate increased to 0.24 d^−1^ for *C. vulgaris* 211-11b and 0.26 d^−1^ for the local isolate (Table 2).

The results of the present work were comparable with previous findings for *C. vulgaris* cultivated in 50% dairy digestate, resulting in a biomass concentration of 2.8 g L^−1^ and biomass productivity of 0.4 g L^−1^ day^−1^, while the highest growth rate was observed in 25% loading [37]. In addition, when 10% agro-waste digestate was used as a substrate, the biomass yield, maximum biomass productivity and maximum growth rate reached 0.57 g L^−1^, 0.032 g L^−1^ day^−1^ and 0.161 day^−1^, respectively [38]. Compared to previous studies, the growth rate of *Chlorella vulgaris* was slightly higher in 10% municipal wastewater digestate enriched with cheese whey, 0.84 d^−1^, while the biomass concentration reached 1.24 g L^−1^ during cultivation in 100% digestate derived from food waste and nappy hydrolysate [39].

### 2.3. Pigments

Pigments represent valuable microalgal compounds used in autotrophic metabolism and photosynthesis. In the present study, chlorophyll a, chlorophyll b and carotenoids were spectrophotometrically estimated throughout the cultivation of both *Chlorella* strains. Pigment concentrations on the 12th and 24th days of growth under batch cultivation in digestate with different initial NH_4_-N contents are listed in Table 3. Pigment production appeared to be affected by the NH_4_-N concentration, but also the light provided in each culture should be taken into consideration. After ammonia stripping, the digestate became slightly less dark and part of its turbidity was possibly lost. As a result, cells in cultures with the pretreated substrate were probably exposed to greater irradiance. The correlation between digestate color and light intensity was studied in more detail by Marcilac et al. [40]. A high concentration of pigments was observed at the end of growth, the static phase, as initially microalgae were consuming the organic substrate available in the digestate. *C. vulgaris* 211-11b showed high pigment production in the culture with 250 mg NH_4_-N L^−^^1^, reaching 38.5 mg L^−1^ or 6.04% *w*/*w* of dry biomass weight. Similarly, the produced pigments of the local isolate reached a concentration of 65.1 mg L^−1^, equal to 6.02% *w*/*w* of dry biomass weight, without prior ammonia stripping. However, the intracellular pigments declined over time under nitrogen limitation, as shown in Table 3. Total chlorophylls and carotenoids of *C. vulgaris* reached 14.2 and 12.9 μg L^−1^, respectively, in mixotrophic cultures with centrate wastewater, under illumination of 10–15 μmol photons m^−2^ s^−1^ [41]. Higher radiation (100 μmol photons m^−2^ s^−1^) for long periods caused a decrease in chlorophyll a, while it enhanced the accumulation of β-carotene in *C. vulgaris* cultivated in a synthetic medium [42]. Moreover, radiation up to 220 μmol photons m^−2^ s^−1^ was applied in batches to determine the dependence of chlorophyll on the NH_4_-N concentration. Under this applied radiation intensity, digestate derived from food waste, containing 400 mg NH_4_-N L^−1^, was used for the cultivation of *C. vulgaris*, which resulted in 28 mg chlorophyll L^−1^. By increasing the NH_4_-N levels to 1000 mg L^−1^, chlorophyll a decreased to 14.9 mg L^−1^ after 7 days of growth [43].

### 2.4. Biomass Composition

**Figure 2:**
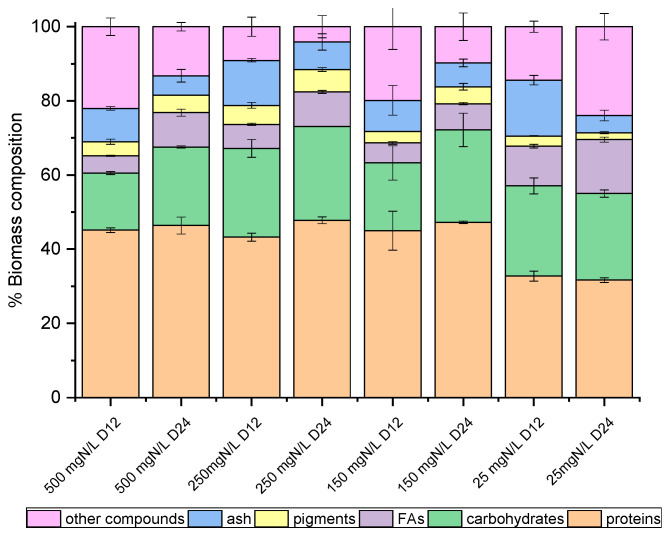
Biomass composition (%) of *C. vulgaris* 211-11b, on dry weight basis, as determined on the 12th and 24th days of growth.

**Figure 3:**
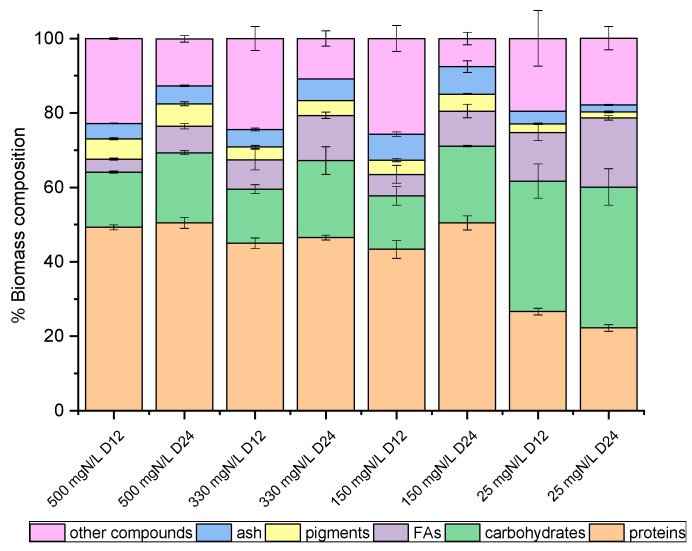
Biomass composition (%) of *Chlorella* sp. (local isolate), on dry weight basis, as determined on the 12th and 24th days of growth.

On the 12th and the 24th days of growth (Figure 1), the produced biomass was harvested and lyophilized in order to determine potential differences in the main intracellular components between the late exponential (12th day) and mature (24th day) phase, with the prospect of investigating potential exploitation routes. As shown in Figure 2 and Figure 3, both species mainly consisted of proteins, the formation of which appeared to be favored by the high levels of NH_4_-N. The proteins of both strains approached 50% *w*/*w* of dry biomass weight under a high NH_4_-N concentration but decreased to 30% *w*/*w* in cultures with only 25 mg NH_4_-N L^−1^. As shown in previous studies, proteins in *C. vulgaris* were close to 50%, carbohydrates were almost 20% and lipids did not exceed 10% of dry cell weight [44]. Based on the results of the present study, when nitrogen became limiting, the accumulation of intracellular carbohydrates and lipids was enhanced. Lipids of both strains were increased between the 12th and 24th days of cultivation, regardless of the initial NH_4_-N content; however, different increment rates were observed between cultures. In particular, the most remarkable lipid production observed was 18.57% *w*/*w* in the case of the local isolate on the 24th day of the nitrogen-limited culture. In contrast, proteins reached up to 60%, while carbohydrates ranged between 25% and 35%, and lipids were only 3% upon nutrient consumption in swine digestate [45]. However, carbohydrates increased against proteins, and fatty acids reached 16.3% under limiting conditions [45]. Lastly, small percentages of the dry biomass were determined as inorganic matter, referred to as ash.

### 2.5. Fatty Acid (FA) Content and Composition Profile

**Figure 4:**
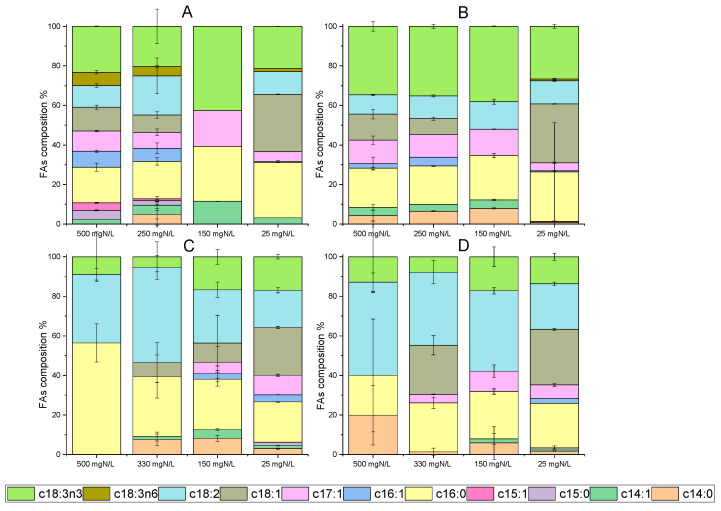
Identification of fatty acids (FAs) in dry biomass of *C. vulgaris* 211-11b and *Chlorella* sp. (local isolate) on the 12th and 24th days of growth. Data are given as percentages (%) of total fatty acids detected ((**A**) FAs for *C. vulgaris* 211-11b on the 12th day, (**B**) FA composition for *C. vulgaris* 211-11b on 24th day, (**C**) FA composition for *Chlorella* sp. (local isolate) on 12th day, (**D**) FA composition for *Chlorella* sp. (local isolate) on 24th day).

Fatty acids from microalgae have great potential for a variety of applications, including in the food industry, nutraceutics and biofuel production [46]. In particular, *C. vulgaris* has given remarkable results, regarding cellular lipid content, simultaneously with wastewater processing. Indicatively, the lipid accumulation reached 37% of dry weight, during cultivation under semi-continuous mode with the addition of poultry compost [47]. Moreover, fatty acids up to 24.5% were noted in batch cultures using diluted agro-waste digestate [38]. Concerning the results of the present study, lipid accumulation under different growth conditions is depicted in Figure 4. Microalgal growth in digestate with different NH_4_-N L^−1^ after ammonia stripping verified that nutrient limitation led to an increase in FAs per dry biomass weight. Palmitic (C16:0), oleic (C18:1) and linoleic (C18:2) acids were mainly detected. By comparing the two strains, *C. vulgaris* 211-11b exhibited high accumulation levels of alpha-linoleic acid (C18:3n3) (Figure 4A,B), while the local isolate presented elevated percentages of linoleic acid (C18:2) (Figure 4C,D). Both alpha-linoleic and linoleic acid occasionally reached 40% of the total fatty acids detected. Palmitic acid was found in large amounts in both microalgal cells. Under nitrogen depletion, palmitic acid accounted for 3.6% of *C. vulgaris* 211-11b dry weight and reached 4.2% in the case of the local isolate. In Figure 4B,D, the above percentages correspond to 25% and 22.5% of total fatty acids for the *C. vulgaris* 211-11b and the local isolate, respectively. Similar to these results, the above fatty acids are listed as some of the most usual in *C. vulgaris* biomass [44,48].

## 3. Materials and Methods

### 3.1. Anaerobic Digestion Effluent

The digestate used in this study originated from the anaerobic digestion of a mixture of expired food products and hydrolysate of used disposable nappies, performed at pilot scale. Anaerobic digestion took place at mesophilic conditions (37 ± 2 °C), in two-stage Continuous Stirred-Tank Reactors (CSTR), where the acidification tank was operating at HRT of 2 days, while, for methanogenesis, HRT was retained at 20 days [49]. After digestate collection, the pretreatment followed included centrifugation (Z 32 HK, Hermle AG, Gosheim, Germany) at 4500 rpm for 7 min and filtration through Whatman glass microfiber filters, GF/F, in order to valorize the solid part for compost production [50] and the liquid part for microalgae cultivation.

### 3.2. Chlorella Strains and Cultivation Conditions

**Figure 5:**
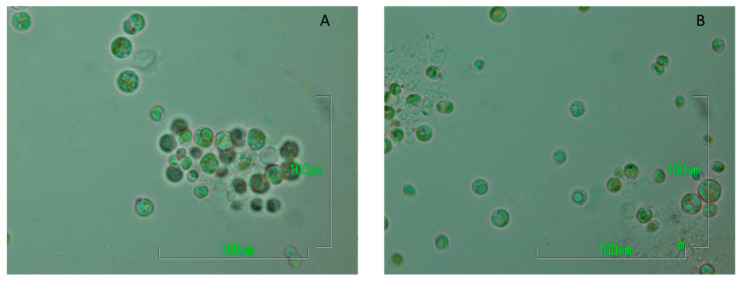
Pictures of the two *Chlorella* strains using the laboratory optical microscope Nikon ECLIPSE E200 (magnification: x100/1.25) ((**A**) picture of *C. vulgaris* 211-11b from a storage culture, (**B**) picture of the Algerian isolate *Chlorella* sp. from a storage culture).

For the present study, two different microalgal strains were used (Figure 5), including *Chlorella vulgaris* (211-11b), obtained from the SAG Culture Collection (University of Göttingen), and a local Algerian isolate of the same genus [51]. Both strains were kept in BG-11 (73816, Sigma-Aldrich) enriched with trace metal solution (Mix A5 with Co 92942, Sigma-Aldrich). Storage cultures were at room temperature (25 °C), while fluorescent light of approximately 25 μmol photons m^−2^ s^−1^ was provided. Prior to inoculation in digestate, pre-cultures of 400 mL total volume, in BG-11 with trace metal solution supplementation, were developed at 25 °C under cool white fluorescent illumination of 200 μmol photons m^−2^ s^−1^. Furthermore, in order to supply CO_2_, atmospheric air was provided at a flow rate of 0.5 L min ^−1^. After 7 days of biomass growth, the microalgal cells were harvested, centrifuged and inoculated in photobioreactors loaded with pretreated digestates. The initial biomass concentration was defined by the determination of the total chlorophylls with a concentration of 1.5 mg L^−1^.

### 3.3. Photobioreactors and Experimental Conditions

Microalgal cultures took place in lab-scale photobioreactors (PBRs), made from Schott-type glass. The photobioreactors, of 1.2 L working volume, were operated at batch mode, under continuous stirring of 250 rpm. In addition, air supply was provided at a rate of 0.5 L min^−1^ by air pumps, while LED strips were attached around the cylindrical reactors to provide 40 μmol photons m^−2^ s^−1^. Both temperature and pH were adjusted at 25 ± 2 °C and 7.8 ± 0.2, respectively, by an automatic controller (sc200, Hach). Undiluted digestate was used as a substrate for all the experimental scenarios, after ammonia stripping pretreatment. Four different concentrations of ammonium nitrogen were tested for each microalgal strain studied. Sampling was carried out daily and then every 2 or 3 days in order to construct biomass growth graphs and estimate the maximum specific growth rate and productivity. However, adequate volumes of sample for biomass characterization were taken on the 12th day of growth to determine the biochemical composition after exponential cell growth and then the mature biomass was collected in its entirety on the 24th day.

### 3.4. Digestate Pretreatment through Ammonia Stripping

Lab-scale ammonia stripping was carried out inside a plexiglass tube of 34 cm height and 8 cm inner diameter. Moreover, 1 L digestate was processed each time, under aeration flow of approximately 2.5 L min ^−1^. The temperature remained at 40 °C via heat exchange between warm water and digestate through the plexiglass cylindrical surface, and the pH value was initially regulated at 12 by adding 6 N NaOH. Notably, the duration of the process ranged from a few hours to a few days, depending on the desired ammonia removal.

### 3.5. Analytical Methods

#### 3.5.1. Biomass Growth

Biomass concentration was estimated as dry cell weight per culture volume, according to *Standard Methods* for the determination of total suspended solids (TSS) [52]. The maximum specific growth rate (μ_max_) was also calculated by the slope of the logarithmic plot of dry weight (DW) as a function of time. Moreover, the productivity of each batch cultivation was estimated as mg L^−1^ day^−1^, based on the slope of the exponential growth, using DW plots versus time.

#### 3.5.2. Microalgal Pigments

Chlorophyll a, chlorophyll b and total carotenoids of wet biomass harvested through centrifugation were estimated after extraction using N, N’-dimethylformamide (DMF) at 25 °C for 20 min. Absorbance at four different wavelengths (480 nm, 646.3 nm, 663.8 nm and 750 nm) was determined spectrophotometrically (Varian Cary 50) and subsequently used according to the available equations [53]. The concentration of chlorophylls in green microalgae cultures is considered to be proportional to the biomass concentration and, at the same time, pigments are an indication of cell vitality [54].

#### 3.5.3. Nutrients and COD Analysis

COD and nutrient determination were performed both for digestate characterization and during microalgal cultivation, in order to evaluate digestate remediation, using filtered samples (Whatman GF/F). Organic carbon, estimated through the Chemical Oxygen Demand (COD) assay, ammonium nitrogen (NH_4_-N), total phosphorous (TP), total solids (TS), volatile solids (VS), total suspended solids (TSS) and volatile suspended solids (VSS) were measured according to *Standard Methods* [52], while the detailed methods can be found in older studies related to the production and processing of digestates [55].

#### 3.5.4. Biomass Composition

After the biomass had been collected from the PBRs and washed with distilled water, it was lyophilized (Telstar, LyoQuest), and the intracellular proteins, carbohydrates, lipids and inorganic components (determined as % ash, free from VSS) were estimated. Biomass proteins were defined through the semi-micro Kjeldahl method [52], quantifying total Kjeldahl nitrogen (TKN) and using a conversion to protein factor of 6.25 [56,57]. The carbohydrates were estimated by the phenol–sulfuric acid assay [58]. Lipid analysis was based on the conversion of fatty acids (FAs) into fatty acid methyl esters (FAMEs) via in-situ transesterification, as previously described [59]. Analysis of FAMEs was performed by gas chromatography (Agilent Technologies, 7890A), where samples were delivered through a capillary column (DB-WAX, 10m × 0.1 mm × 0.1 μm) to a Flame Ionization Detector (FID). For the analysis, temperature reached 230 °C in the column and 250 °C at both the injector and the detector. Helium was used as a carrier gas with average velocity of 30.3 cm s^−1^. To quantify the produced FAMEs, a reference standard (FAMQ-005, Accustandard) and an internal standard solution (C17: 0, Sigma) were used. Lastly, the percentage of ash was determined gravimetrically through the vs. assay [52], while the total biomass composition was calculated on a dry weight basis, by taking into consideration the moisture content that was measured through the TS assay [52].

#### 3.5.5. Statistical Analysis

One-way Analysis of Variance (ANOVA) with *p* value equal to 0.05 was carried out in order to understand how the initial NH_4_-N concentration affected the performance of the two *Chlorella* strains. The *Minitab 19* software was used to statistically group the results after Tukey’s test (*p* = 0.05).

## 4. Conclusions

Cultivation of microalgae in digestates is a potentially effective digestate bioremediation method. However, high nitrogen levels often necessitate a pretreatment step prior to microalgal cultivation. In the present study, ammonia stripping was applied, as an easily applicable and low-cost nitrogen recovery method, followed by microalgal growth. Although both *Chlorella* strains exhibited satisfactory adaptability and growth in all the initial NH_4_-N concentrations tested, results were improved by reducing the digestate’s nitrogen content. Lastly, the Algerian isolate of *Chlorella* sp. showed a remarkable biomass composition, while, besides high protein content, intracellular carbohydrates and fatty acids were also accumulated, with potential added-value applications. Although total phosphorus was completely removed in all cultures, NH_4_-N was fully consumed only in media with an initial concentration of 25 mg NH_4_-N L^−1^. The local isolate exhibited higher biomass production than *C. vulgaris* 211-11b in untreated digestate (1.07 against 0.63 g L^−1^) and showed the highest fatty acid content (18.57% *w*/*w*) under nitrogen-limiting conditions.

## Figures and Tables

**Table 1:** Digestate remediation, in terms of COD, NH_4_-N and TP removal, for each strain and initial ammonium nitrogen concentration examined. Data are presented for the beginning and the end of cultivation, as means ± SD (*n* = 2).

		*C. vulgaris* (211-11b)				*Chlorella* sp. (Local Isolate)			
	(mg L^−1^)	500 mg L^−1^ NH_4_-N	250 mg L^−1^ NH_4_-N	150 mg L^−1^ NH_4_-N	25 mg L^−1^ NH_4_-N	500 mg L^−1^ NH_4_-N	330 mg L^−1^ NH_4_-N	150 mg L^−1^ NH_4_-N	25 mg L^−1^ NH_4_-N
COD	Initial conc.	3195.7 ± 112.9	2616.5 ± 39.5	2783.7 ± 120.2	1510.0 ± 5.0	3493.6 ± 20.5	2837.0 ± 72.8	2297.0 ± 63.6	1176.1 ± 46.9
	Removal (%)	90.8 ± 4.8 ^a^	82.2 ± 3.5 ^a^	86.5 ± 5.7 ^a^	57.5 ± 0.3 ^b^	89.6 ± 1.2 ^a^	84.6 ± 3.4 ^a^	85.2 ± 4.2 ^a^	58.2 ± 4.8 ^b^
NH_4_-N	Initial conc.	499.0 ± 1.4	247.0 ± 1.4	145.0 ± 4.1	23.7 ± 0.4	487.0 ± 5.7	324.0 ± 9.9	147.8 ± 9.5	24.7 ± 0.3
	Removal (%)	19.7 ± 0.8 ^e^	39.1 ± 3.0 ^d^	67.2 ± 5.4 ^b,c^	96.2 ± 2.7 ^a^	39.7 ± 2.3 ^d^	50.5 ± 3.6 ^c,d^	81.9 ± 11.1 ^a,b^	98.4 ± 1.7 ^a^
TP	Initial conc.	9.2 ± 0.5	7.8 ± 0.3	7.7 ± 0.2	12.6 ± 0.1	12.9 ± 1.0	11.1 ± 1.4	10.0 ± 0.9	10.1 ± 1.0
	Removal (%)	88.0 ± 9.9	100.0 ± 8.6 ^a^	93.5 ± 3.6 ^a^	65.9 ± 2.4 ^a^	92.2 ± 10.5 ^a^	89.2 ± 17.6 ^a^	91 ± 12.9 ^a^	88.1 ± 13.2 ^a^

Means that do not share a letter are significantly different.

**Table 2:** Biomass production (g DW L^−1^), maximum growth rate (μ_max_, d^−1^) and exponential growth phase productivity (g L^−1^ d^−1^) of *C. vulgaris* 211-11b and the local isolate under different initial NH_4_-N levels.

	*C. vulgaris* (211-11b)				*Chlorella* sp. (Local Isolate)			
	500 mg L^−1^ NH_4_-N	250 mg L^−1^ NH_4_-N	150 mg L^−1^ NH_4_-N	25 mg L^−1^ NH_4_-N	500 mg L^−1^ NH_4_-N	250 mg L^−1^ NH_4_-N	150 mg L^−1^ NH_4_-N	25 mg L^−1^ NH_4_-N
Biomass Conc. (g L^−1^)	0.63 ± 0.04 ^c^	0.69 ± 0.05 ^c^	0.86 ± 0.06 ^b,c^	1.02 ± 0.14 ^a,b,c^	1.07 ± 0.06 ^a,b,c^	1.34 ± 0.18 ^a,b^	1.38 ± 0.2 ^a^	1.43 ± 0.10 ^a^
μ_max_ (day^−1^)	0.23 ± 0.00 ^a,b^	0.24 ± 0.02 ^a,b^	0.19 ± 0.08 ^a,b^	0.15 ± 0.01 ^a,b^	0.26 ± 0.03 ^a^	0.25 ± 0.00 ^a,b^	0.24 ± 0.00 ^a,b^	0.13 ± 0.02 ^b^
Productivity (g L^−1^ d^−1^)	0.08 ± 0.00 ^a,b^	0.10 ± 0.01 ^a,b^	0.07 ± 0.02 ^b^	0.07 ± 0.00 ^b^	0.12 ± 0.00 ^a,b^	0.13 ± 0.03 ^a^	0.10 ± 0.00 ^a,b^	0.08 ± 0.00 ^a,b^

Means that do not share a letter are significantly different.

**Table 3:** Concentration of pigments, chlorophylls a, b (C_a+b_) and total carotenoids (Crt), in *C. vulgaris* 211-11b and the local isolate cultures. Outset concentrations of chlorophylls a, b and total carotenoids were 1.5 and 0.3 mg L^−1^, respectively, for all initial NH_4_-N concentrations.

	*C. vulgaris* (211-11b)								*Chlorella* sp. (Local Isolate)							
	500 mg L^−1^ NH_4_-N		250 mg L^−1^ NH_4_-N		150 mg L^−1^ NH_4_-N		25 mg L^−1^ NH_4_-N		500 mg L^−1^ NH_4_-N		250 mg L^−1^ NH_4_-N		150 mg L^−1^ NH_4_-N		25 mg L^−1^ NH_4_-N	
(mg L^−1^)	C_a+b_	Crt	C_a+b_	Crt	C_a+b_	Crt	C_a+b_	Crt	C_a+b_	Crt	C_a+b_	Crt	C_a+b_	Crt	C_a+b_	Crt
Day 12	21.5 ± 2.9 ^c,d^	3.7 ± 0.4 ^C^	30.4 ± 0.0 ^b,c^	5.1 ± 0.1 ^A,B,C^	19.3 ± 0.3 ^d^	5.1 ± 0.2 ^A,B,C^	19.5 ± 2.8 ^c,d^	3.7 ± 0.5 ^C^	45.2 ± 7.4 ^a^	7.4 ± 1.1 ^A^	33.5 ± 0.1 ^b^	4.8 ± 0.2 ^A,B,C^	39.4 ± 0.5 ^a,b^	7.3 ± 1.7 ^A,B^	21.2 ± 0.0 ^c,d^	4.3 ± 0.0 ^B,C^
Day 24	26.5 ± 3.6 ^c,d,e^	3.7 ± 0.2 ^D,E^	38.5 ± 1.8 ^b,c^	5.2 ± 0.3 ^C,D^	33.2 ± 3.8 ^c,d^	7.6 ± 1.2 ^B^	16.6 ± 0.5 ^e^	2.8 ± 0.5 ^E^	65.1 ± 6.0 ^a^	11.1 ± 0.4 ^A^	52.7 ± 4.6 ^a,b^	6.4 ± 0.8 ^B,C^	57.8 ± 3.7 ^a^	12.7 ± 0.1 ^A^	19.1 ± 1.1 ^d,e^	4.3 ± 0.1 ^C,D,E^

Means that do not share a letter are significantly different.

## Data Availability

Not applicable.
